# Isolation of the isopropanol extract components of the leaves of *Ailanthus glandulosa* by a new method

**DOI:** 10.1016/j.heliyon.2023.e15881

**Published:** 2023-04-30

**Authors:** Sawsan Youseff Saad

**Affiliations:** Faculty of Dentistry, Manara University, Lattakia, Syria

**Keywords:** Ailanthus glandulosa, Isopropanol extract, Mass spectrometry, Column chromatography

## Abstract

The leaves of *Ailanthus glandulosa* were extracted with the Soxhlet apparatus using isopropanol. A new method was used to separate and isolate eleven chemical compounds present in the leaves of bird's tongue. The separation was carried out by column chromatography using displacement solvents (petroleum ether, chloroform, dichloromethane, methanol) and four eluates were obtained. The four eluates were treated with a number of solvents, yielding thirty-four compounds. The chemical content of the mordants was determined using GC/MS technology. The samples tested contained six ester compounds, three aldehyde compounds, three ketone compounds, two alcoholic compounds, eight carboxylic acid compounds, five silicone compounds, five aromatic compounds and one phosphate compound.

Eleven compounds were isolated, the most important of which are: 2-naphthoxyacetic acid, 2,6-bis (1,1-dimethylethyl)-4-ethylphenol, 2,5-*tert*-butylnitrobenzene, 5-hexyl-2-furaldehyde, 16-nitrobicyclo [10.4.0] hexadecan-1-ol-13-one, cyclooctasiloxan hexadecamethyl.

## 1 Introduction

*Ailanthus glandulosa*, scientific name: *Ailanthus altissima*. It belongs to the samrobiate family and is a widespread deciduous forest tree found in temperate climates on both sides of roads, in wastelands and in abandoned areas. Its leaves are compound, green in colour and fall off in winter [1]. It belongs to the genus *Ailanthus* in the family Simarobica. It is called by many names depending on the country of origin, such as Tree of Heaven, Chinese Sumac, Copal Tree and Brooklyn Palm, Hell Tree. In the Middle East, this tree is known as Chinese Azdoracht or Chinese Azurekh. It is native to north-eastern and central China and Taiwan [2]. In many areas it is called the tree of hell because it grows very fast and can clone itself.

In America and Europe it is considered an invasive plant. It has medicinal and biological properties [[1], [2], [3], [4], [5], [6]].

Researchers have demonstrated the effectiveness of the methanol extract from the leaves of *Ailanthus glandulosa* against free radicals (antioxidant) [7]. Some studies have also shown that the aqueous extract from the leaves of the *Ailanthus glandulosa* tree has antioxidant properties [8]. One study showed that the bark extract with petroleum ether had a strong repellent and killing effect against four major insects in shops and warehouses [9]. Other researchers studied the chemical constituents of the bark of the *Ailanthus glandulosa* tree and were able to isolate six canthinone-type alkaloids, also investigating the effectiveness of these constituents as anti-inflammatory [10].

Researchers synthesized copper oxide nanoparticles (ZnO-NPs) using the aqueous extract from the fruits of the *Ailanthus glandulosa* tree, then studied the effect of secondary copper oxide on some bacteria and concluded that this compound can be used as an antibacterial agent [11]. The researchers showed that the methanolic extracts of the leaves have the highest content of total phenols and have high antioxidant properties. They found that the acetone, methanol and dichloromethane extracts were very effective against Gram-positive bacteria but not against Gram-negative bacterial strains [12]. Researchers studied the chemical composition of *Ailanthus glandulosa* extract with chloroform as a solvent and demonstrated its toxic effect on whiteflies [13].

Plant extracts are currently a broad field for study due to their diversity and different uses. The aim of the research was therefore to: search for a new method to isolate the constituents of the leaves of *Ailanthus glandulosa* using a new solvent not previously used in reference studies, isopropanol, and investigate the chemical structures of the isolated compounds using GC/MS.

## 2 Experimental

### 2.1 General information

The required chromatograms were obtained with a gas chromatography coupled GC/MS instrument of the SHIMADZU type, model GCMS-QP2010 plus, using a capillary column of the type (TRB-WAX) with dimensions (length 60 m * diameter 0.25 mm * thickness 0.25 μm) and a carrier gas containing helium with a purity of 99.9999. %. The mass spectra were recorded from *m*/*z* 15 to *m*/*z* 450.

Chemical and starting materials: The solvents used were from different companies (Sigma Aldrich, Merck, Fluka, BDH) (methanol, hexane, ethyl ether, chloroform, dichloromethane, petroleum ether …).

### 2.2 Extract

The leaves of *Ailanthus glandulosa* were collected in June of 2021 in Lattakia Governorate, Syria. The leaves were thoroughly cleaned of dust and suspended impurities, dried in a well-ventilated, shaded place at room temperature (20–25 °C) for about one month, then crushed to obtain the required fineness.

The extract was obtained using a Soxhlet apparatus by adding 200 g of the crushed dried leaves into the thimble and then adding 1000 ml of isopropanol into the 2000 ml capacity flask of the apparatus. The extraction process took 72 h, after which the extract was concentrated using a rotary evaporator. A green, viscous extract was obtained at a temperature of 40 °C to a volume of 5 ml. Separation of the extract by column chromatography with the solvents (petroleum ether, dichloromethane, chloroform, methanol), yielding four eluates. The solvents of the four eluates were evaporated with a rotary evaporator at a temperature of 40 °C.For the first time, a new method was used to separate and isolate a number of chemical compounds from the leaves of *Ailanthus glandulosa*.

1. Petroleum eluate (A): It has an olive green colour.
2. Dichloromethane eluate (B): The product was dissolved with methanol and then chloroform was added to form a precipitate in the form of a mixture of a white and a brown precipitate. The precipitate formed was dissolved again with methanol, then chloroform was added to the solution and a white precipitate and a brown filtrate were formed. The filtrate was treated with the following solvents: 2-methylbutane, pentane, chloroform, methanol, hexane, dichloromethane and diethyl ether, and a series of samples was obtained.
3. Chloroformeluate (C): Eluate C was treated as follows:
  a Using 2-methylbutane and chloroform, sample C_1_ was obtained.
  b Using methanol and pentane, both samples C_2_ were obtained.
  c Using methanol and dichloromethane, sample C_3_ was obtained.
  d Samples C_m_ and C_n_ were isolated from sample C_2_ using diethyl ether and methanol.
  e Sample C_3t_ was isolated using methanol and ethyl ether.
4. Methanol eluate (D): Eluate D was treated with methanol and chloroform in different ratios (2:1, 1:1, 1:2, 1:3, 1:4, 2:5) and we obtained samples (D_1_, D_2_, D_3_, D_4_, D_5_, D_6_, D_7_) respectively.

**Fig. 1:**
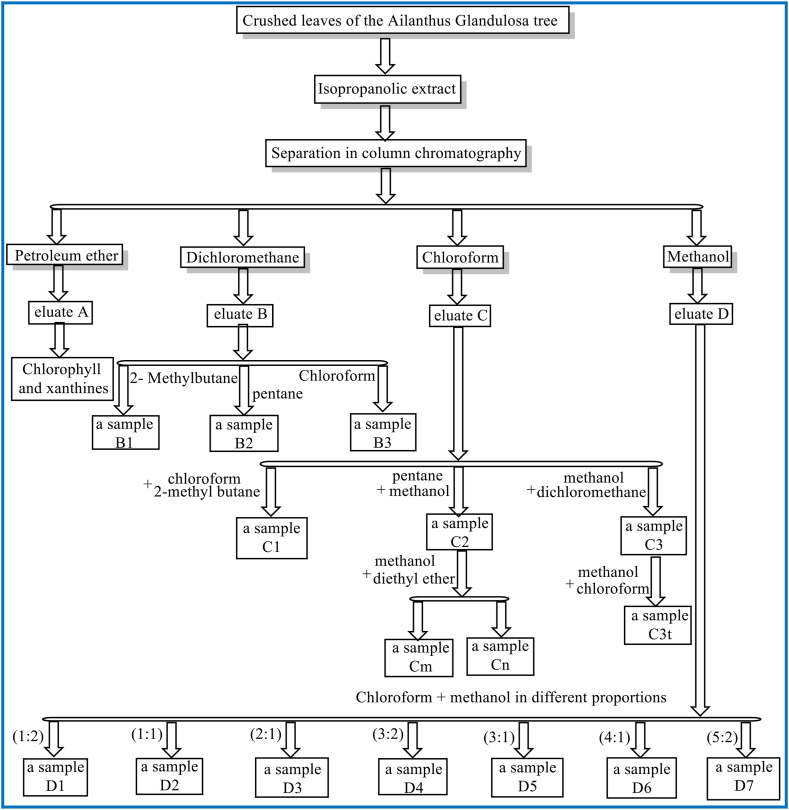
Scheme of work in the extraction and separation of compounds.

Fig. 1 Shows the steps of the previous work.

### 2.3 Organic feed analysis

Analysis was performed by injecting 2 μl of each sample into a device GC-MS at a flow rate of 1 ml/min. The solvent used was chloroform. Then the injector temperature was set at 250Cͦ, ionization source temperature at 225Cͦ and the quadrupole temperature at 130–150Cͦ. The thermal programme started at 200Cͦ, the temperature of the oven column, and this temperature was maintained for 1 min (1 min), then it was increased to 270Cͦ at a rate of 5Cͦ per minute, and this temperature was maintained for 35 min, and the mass spectra were recorded from 15 m\z to 450 m\z. The chemical compounds present in each pathway of the *Ailanthus glandulosa* leaf extract were determined by comparing the mass spectra generated for each peak with the mass spectra of the compounds in the HP Mass Sperliral Library Nist Wiley. The fragmentation mass spectra of some compounds were examined to confirm their structures based on the fragmentation mechanism shown in the recorded diagrams.

## 3 Results and discussion

The weight of the resulting isopropanolic extract was (4 g).

This extract was treated with some solvents and separated into eluates containing fewer compounds by column chromatography. This makes it easier for us to examine the chromatograms of these eluates and accurately identify the components of the extract.

**Fig. 2:**
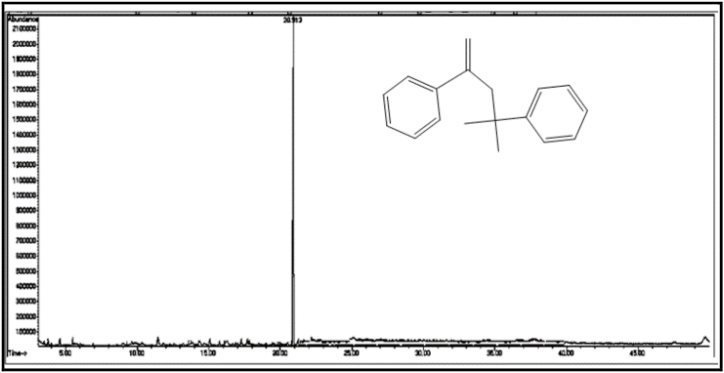
GC/MS chromatogram of sample B_1_ from dichloromethane eluate.

**Fig. 3:**
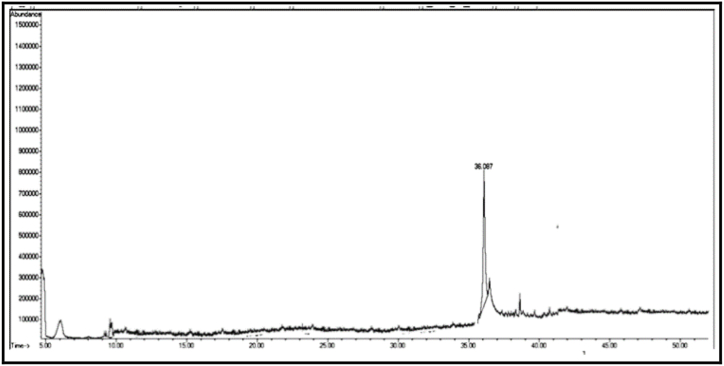
GC/MS chromatogram of sample B_2_ from dichloromethane eluate.

**Fig. 4:**
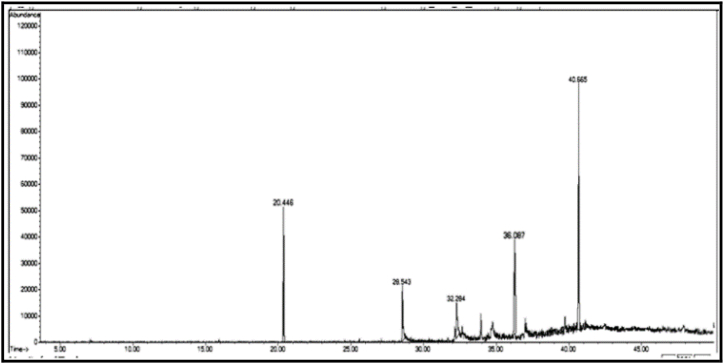
GC/MS chromatogram of sample B_3_ from dichloromethane eluate.

1 The chemical compounds contained in the petroleum ether eluate (eluate A): It contains chlorophyll and xanthates.
2 The chemical compounds contained in the eluate dichloromethane (eluate B):

The GC/MS chromatogram of sample B_1_ (Fig. 2) shows the presence (99.98%) of a single compound, 2,4-diphenyl-4-methyl-1-pentene. The GC/MS chromatogram of sample B_2_ (Fig. 3.) also shows the presence of a single compound, 1,3,3-trimethyl-1-phenylindane (98.01%), indicating good separation in column chromatography. The GC/MS chromatogram of sample (B_3_) (Fig. 4) shows the presence of five ester compounds accounting for (97.87%) of the total sample. The compound with the highest percentage in the sample is 1,2-benzenedicarboxylic acid diisooctyl ester (41.14%), and the compound with the lowest percentage is di-ethylhexyl phthalate (3.21%).

**Fig. 5:**
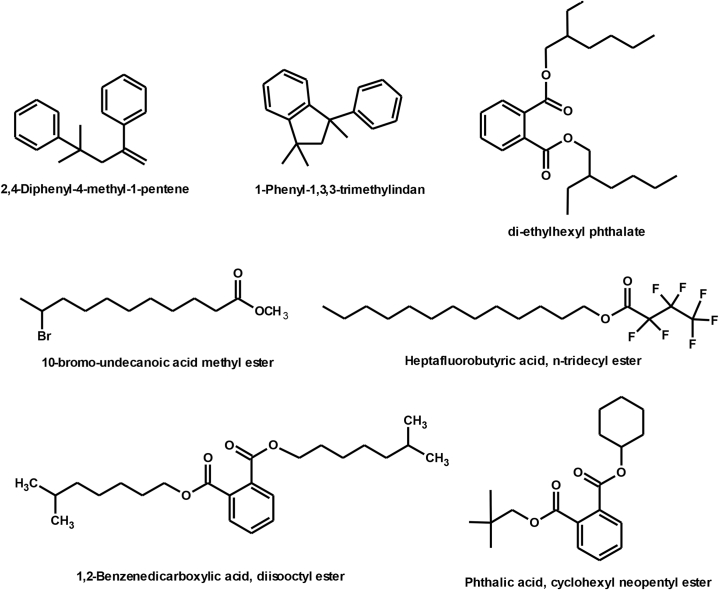
Formulas of some chemical compounds present in the eluate (B).

The chemical structure of some compounds was confirmed by mass spectrometry. Table 1 shows the chemical constituents of the samples (B_1_, B_2_, B_3_) of dichloromethaneluate. Fig. 5 shows the formulae of some chemical compounds contained in the samples of eluate B.

**Table 1:** The chemical composition of samples (B_1_, B_2_, B_3_) of dichloromethane eluate.

sample code	compound name	Molecular formula	molecular massg/mol	R_t_	relative peak area %
Sample Components (B_1_)					
**B**_**1**_	2,4-Diphenyl-4-methyl-1-pentene	C_18_H_20_	236.4	20.913	99.98
Sample Components (B_2_)					
**B**_**2**_	1,3,3-trimethyl-1-phenyl indane	C_18_H_20_	236.4	36.087	98.01
Sample Components (B_3_)					
**B**_**3a**_	10-bromo-undecanoic acid methyl ester	C_12_H_23_BrO_2_	279.21	20.446	31.13
**B**_**3b**_	Heptafluorobutyric acid,n-tridecylester	C_17_H_27_O_2_F_7_	396.3	28.543	6.08
**B**_**3c**_	di-ethylhexyl phthalate	C_24_H_38_O_4_	390.5	32.284	3.21
**B**_**3d**_	Cyclohexyl neopentyl phthalate	C_19_H_26_O_4_	318.4	36.087	16.31
**B**_**3f**_	1,2-Benzene dicarboxylic acid diisooctylester	C_24_H_38_O_4_	390.5	40.665	41.14
Total					97.87

It was found that there are two aromatic compounds and three compounds belonging to the group of phthalic acid derivatives. These compounds (phthalates) may be of plant origin or due to environmental pollution, as we found them in the isopropanolic extract from the leaves of the *Ailanthus glandulosa* tree.

In the chromatograms of samples B_2_ and B_3_ (Fig. 3, Fig. 4), small peaks were observed that were not detected by the instrument.

**Fig. 6:**
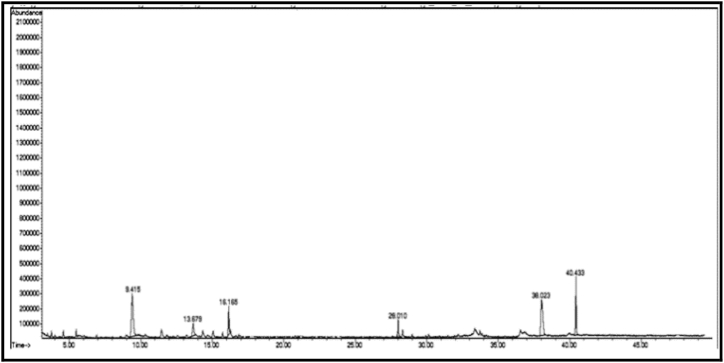
The GC/MS chromatogram of sample (C_1_) from the chloroform eluate.

**Fig. 7:**
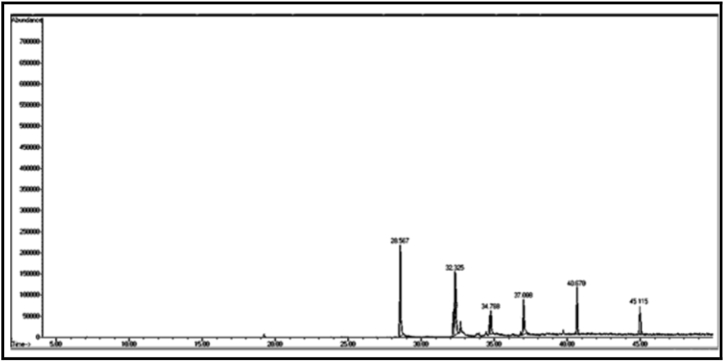
The GC/MS chromatogram of sample (C_2_) from the chloroform eluate.

**Fig. 8:**
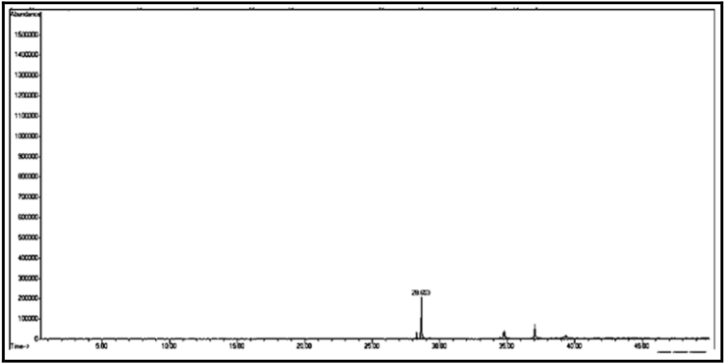
The GC/MS chromatogram of sample (C_m_) from the chloroform eluate.

**Fig. 9:**
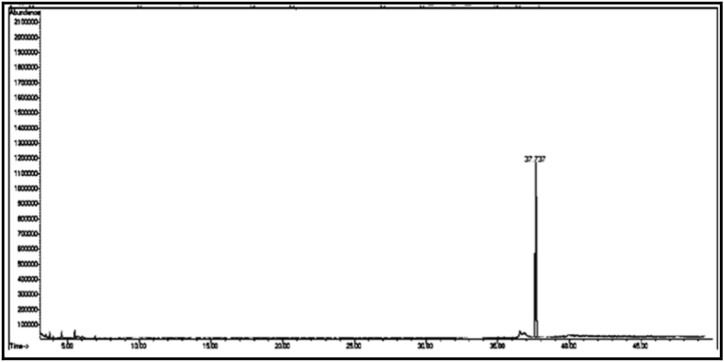
The GC/MS chromatogram of sample (C_n_) from the chloroform eluate.

**Fig. 10:**
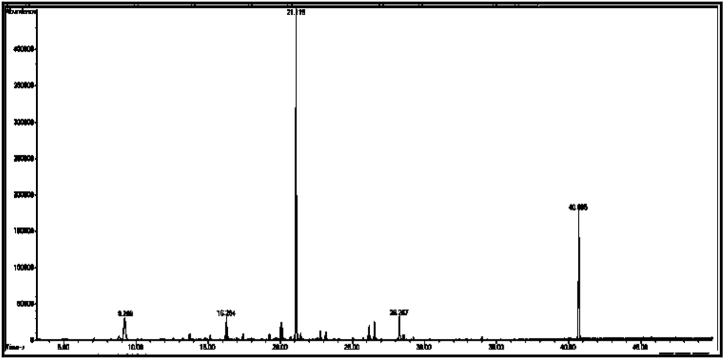
The GC/MS chromatogram of sample (C_3_) from the chloroform eluate.

**Fig. 11:**
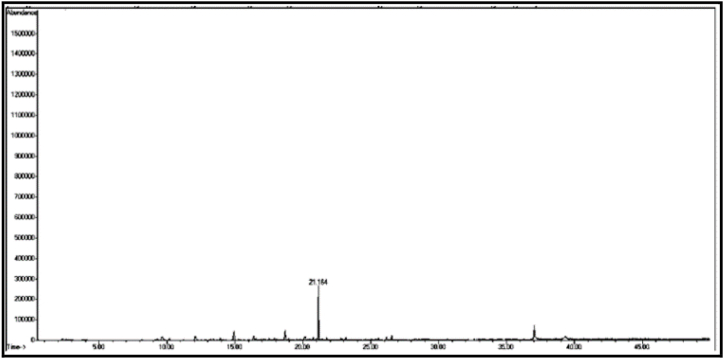
The GC/MS chromatogram of sample (C_3t_) from the chloroform eluate.

3 Chemical compounds present in chloroform (eluate C): Fig. 6, Fig. 7, Fig. 8, Fig. 9, Fig. 10, Fig. 11 show the GC/MS chromatograms of samples C_1_, C_2_ and C_3_ of chloroform eluate.

From the GC/MS chromatogram of sample (C_1_) (Fig. 6), it can be seen that six aldehydes, ketones and alcohols (98.12%) of the total sample are present. The compound with the highest percentage in the sample is 2-methyl-*Z*,*Z*-3,13-octadecadienol (27.10%), while the compound 2-methylundecanal has the lowest percentage (5.15%).

From the GC/MS chromatogram of sample (C_2_) (Fig. 7), it can be seen that the six compounds are carboxylic acids, accounting for 99.75% of the total number of sample (C_2_). The compound with the highest percentage in the sample is: tetradecanoic acid (C_2a_) (29.01%), while the compound heptadecanoic acid (C_2c_) has the lowest percentage (5.10%). Two compounds were isolated from the sample (C_2_), namely (C_m_ and C_n_).

From the GC/MS chromatogram of sample C_m_ (Fig. 8), it can be seen that it is a single compound. After investigating the fragmentation mechanism by mass spectrometry, the compound was found to be 2-naphthoxyacetic acid (C_m_). The GC/MS chromatogram of sample C_n_ (Fig. 9) shows that it is also a single compound. After investigating the fragmentation mechanism by mass spectrometry, the compound was found to be tridecanoic acid (Cn).

The GC/MS chromatogram of sample (C_3_) (Fig. 10) shows the presence of five silicon compounds accounting for 97.38% of the total sample, the compound with the highest percentage being tetradecamethyl cycloheptasiloxane (60.87%). It was also found that there are other compounds with lower percentages than those mentioned above, the most important of which is: octadecamethyl cyclononasiloxane (24.98%). Only one compound C_3t_, was isolated from sample C_3_. It is noted from the GC/MS chromatogram of sample C_3t_ (Fig. 11.) that there is only one compound, and it was found after a mechanical study. Fragmentation by mass spectrometry revealed that the compound is hexadecamethyl cyclooctasiloxane.

Table 2 shows the chemical composition of the samples (C_1_, C_2_, C_3_) from the chloroform eluate.

**Table 2:** The chemical composition of samples (C_1_, C_2_, C_3_) of chloroform eluate.

sample code	compound name	Molecular formula	Mw (g/mol)	R_t_	relative peak area %
Sample Components (C_1_)					
**C**_**1a**_	(*E*,*E*) 2,4-decadienal	C_10_H_16_O	152.2	9.415	22.01
**C**_**1b**_	2-Methylundecanal	C_12_H_24_O	184.3	13.679	5.15
**C**_**1c**_	2-Methyl-2-cyclohexenone	C_7_H_10_O	110.1	16.165	15.98
**C**_**1d**_	1-Methyl-1-pentadecyne, cyclohexene	C_22_H_40_	304	28.010	7.53
**C**_**1f**_	3,5-Dimethyl-2-octanol	C_10_H_22_O	158.28	38.023	20.35
**C**_**1g**_	2-Methyl-*Z*,*Z*-3,13-octadecadienol	C_19_H_36_O	280.5	40.433	27.10
Total					98.12
Sample Components (C_2_)					
**C**_**2a**_	Tetradecanoic acid	C_14_H_28_O_2_	228.3	32.325	29.01
**C**_**2b**_	(Z) 11-Hexadecenoic acid	C_16_H_30_O_2_	254.4	34.768	25.12
**C**_**2c**_	Heptadecanoic acid	C_17_H_34_O_2_	270.5	36.007	5.10
**C**_**2d**_	Octadecanoic acid	C_18_H_36_O_2_	284.4	37.008	11.20
**C**_**2f**_	Nonadicenoic acid	C_19_H_36_O_2_	296.5	40.670	20.11
**C**_**2g**_	Decadecanoic acid	C_20_H_38_O_3_	312	45.115	9.21
Total					99.75
Sample Components (C_m_)					
**C**_**m**_	2- Naphthoxy acetic acid	C_12_H_10_O_3_	202.2	28.653	98.17
Sample Components (C_n_)					
**C**_**n**_	Tridecanoic acid	C_13_H_26_O_2_	214.3	37.737	99.63
Sample Components (C_3_)					
**C**_**3a**_	1,1,1,5,5,5-hexamethyl-3,3-bis [(trimethylsilyl)oxy] trisiloxane	C_12_H_36_O_4_Si_5_	384.83	9.208	4.39
**C**_**3b**_	3-Isopropoxy-1,1,1,7,7,7-hexamethyl-3,5,5-tris (trimethylsiloxy) tetra siloxane	C_18_H_52_O_7_Si_7_	577.2	16.284	3.92
**C**_**3d**_	Tetradecamethyl cycloheptasiloxane	C_14_H_42_O_7_Si_7_	519	21.119	60.87
**C**_**3f**_	Hexadecamethyl cyclooctasiloxane	C_16_H_48_O_8_Si_8_	593	28.267	3.22
**C**_**3g**_	Octadecamethyl cyclononasiloxane	C_18_H_54_O_9_Si_9_	667	40.658	24.98
Total					97.38
Sample Components (C_3t_)					
**C**_**3t**_	Hexadecamethyl cyclooctasiloxane	C_16_H_48_O_8_Si_8_	593	21.164	98.82

**Fig. 12:**
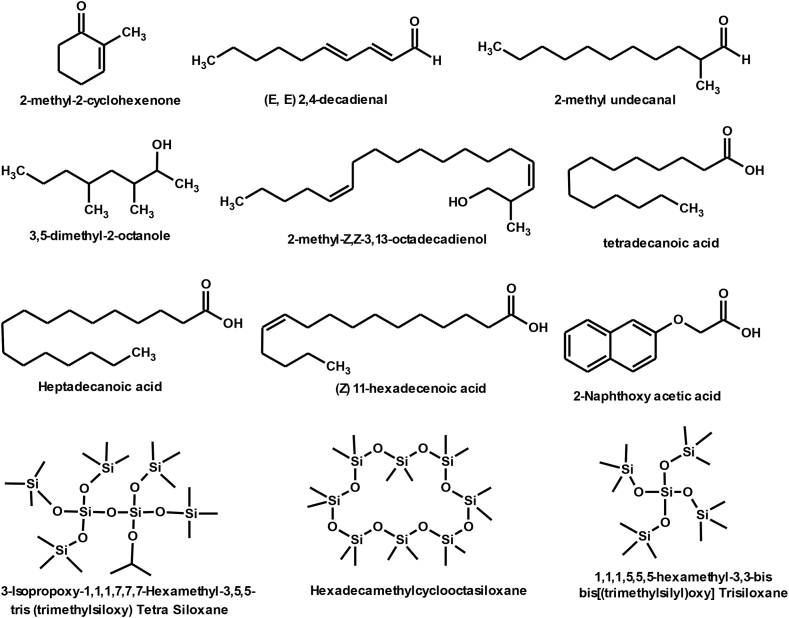
Formulas of some chemical compounds present in eluate samples C.

Fig. 12 Shows the formulae of some chemical compounds in the samples of eluate C. There are small peaks that are not detected by the instrument.

4.The chemical compounds in the methanol eluate (D): The eluate (D) was treated with the following solvents: Methanol and Chloroform, and samples (D_1_-D_7_) were obtained.

**Fig. 13:**
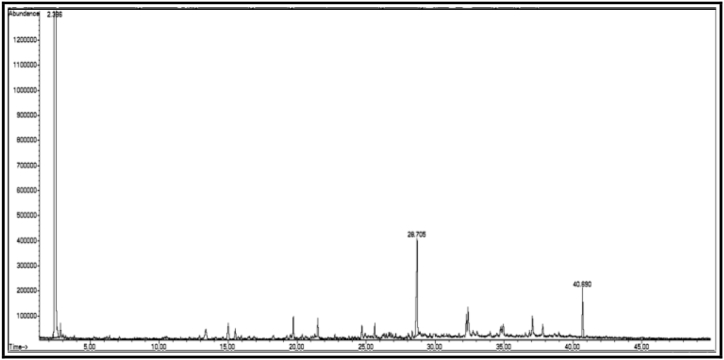
The GC/MS chromatogram of sample (D_1_) from the methanol eluate.

**Fig. 14:**
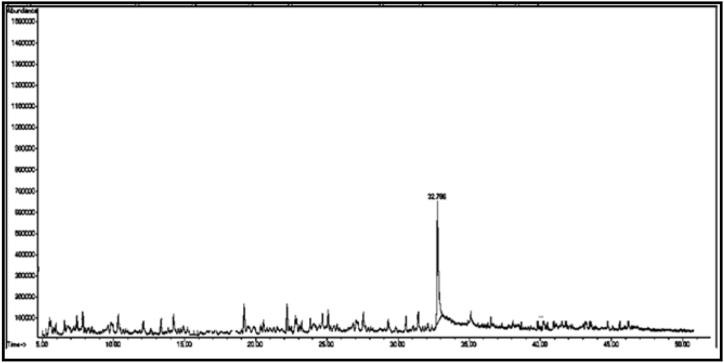
The GC/MS chromatogram of sample (D_2_) from the methanol eluate.

**Fig. 15:**
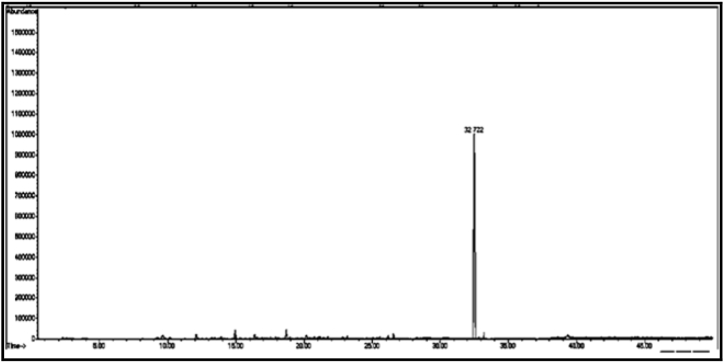
The GC/MS chromatogram of sample (D_3_) from the methanol eluate.

**Fig. 16:**
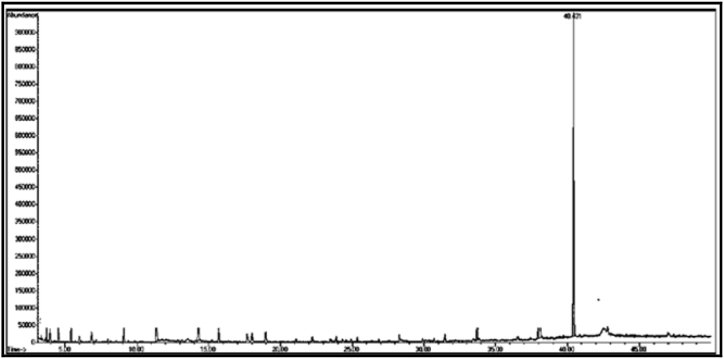
The GC/MS chromatogram of sample (D_4_) from the methanol eluate.

**Fig. 17:**
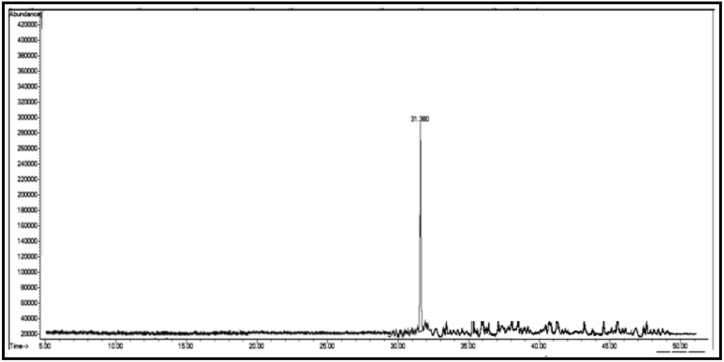
The GC/MS chromatogram of sample (D_5_) from the methanol eluate.

Fig. 13 Shows the GC/MS chromatogram of sample (D_1_), the presence of two compounds representing 89.95% of the total sample, the compound with the highest percentage in the extract is: 2-(2-ethoxyethoxy) -phosphate-ethanol (55.32%). The GC/MS chromatogram of the sample (D_2_) (Fig. 14.) shows that one compound is 2,6-bis(1,1-dimethylethyl)-4-ethylphenol. As can be seen from the GC/MS chromatogram of sample (D_3_) (Fig. 15.), the only compound is 2,5-di-*tert*-butylnitrobenzene, which accounts for 99.81% of the total sample. From the GC/MS chromatogram of sample (D_4_) (Fig. 16), it can be seen that there is also a single compound, 2-allyl-2-methyl-1,3-cyclopentanedione, accounting for (98.34%) of the total amount of the above sample. As can be seen from the GC/MS chromatogram of sample (D_5_) (Fig. 17), the single compound is 2-ethylhexyl chloroformate.

**Fig. 18:**
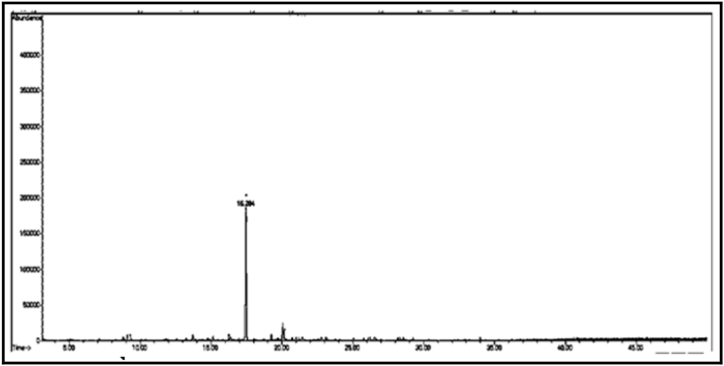
The GC/MS chromatogram of sample (D_6_) from the methanol eluate.

**Fig. 19:**
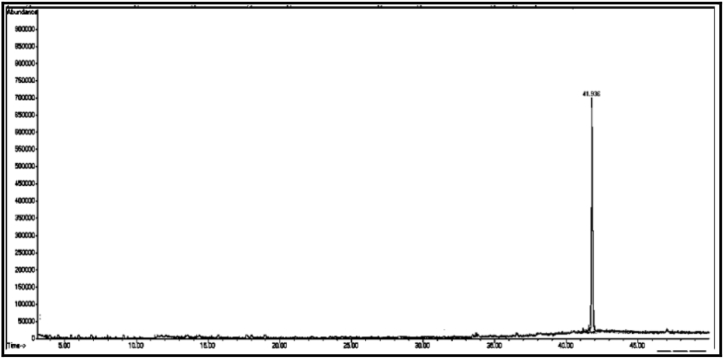
The GC/MS chromatogram of sample (D_7_) from the methanol eluate.

The GC/MS chromatogram of sample (D_6_) in Fig. 18 shows that the single compound is 5-hexyl-2-furaldehyde with a percentage of (99.14%). Fig. 19: The GC/MS chromatogram of sample (D_7_) shows the presence of a single compound, 16-nitrobicyclo [10.4.0] hexadecan-1-ol-13-one, with a percentage of (99.98%).

Table 3 shows the chemical composition of the methanol eluate samples (D eluate).

**Table 3:** The chemical composition of samples (D_1_-D_7_) of methanol eluate.

sample code	compound name	Molecular formula	molecular massg/mol	R_t_	relative peak area %
Sample Components (D_1_)					
**D**_**1a**_	2-(2-Ethoxyethoxy) phosphate ethanol	C_6_H_15_O_6_P	214	28.705	55.32
**D**_**1b**_	5,6,7,7a-Tetrahydro-4,4,7a-trimethylbenzofuran-2(4H)-one	C_11_H_16_O_2_	180.2	40.690	34.63
Total					89.95
Sample Components (D_2_)					
**D**_**2**_	2,6-Bis(1,1-Dimethylethyl)-4-ethyl phenol	C_16_H_26_O	234.38	32.786	57.37
Sample Components (D_3_)					
**D**_**3**_	2,5-Di-*tert*-butylnitrobenzene	C_14_H_21_NO_2_	235.3	32.722	99.81
Sample Components (D_4_)					
**D**_**4**_	2-Allyl-2-methyl-1,3-cyclopentandione	C_9_H_12_O_2_	152.1	40.431	98.34
Sample Components (D_5_)					
**D**_**5**_	2-Ethylhexylchloroformate	C_9_H_17_ClO_2_	192.6	31.380	99.35
Sample Components (D_6_)					
**D**_**6**_	5-Hexyl-2-furaldehyde	C_11_H_16_O_2_	180.2	17.56	99.14
Sample Components (D_7_)					
**D**_**7**_	16-Nitrobicyclo [10.4.0] hexadecan-1-ol-13-one	C_16_H_27_NO_4_	297.3	41.806	99.98

**Fig. 20:**
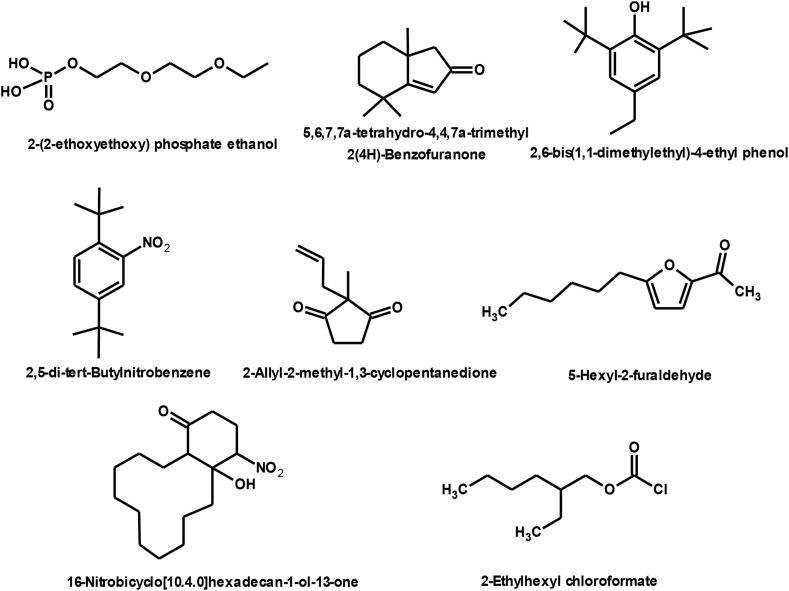
Formulas of some chemical compounds present in methanol eluate.

Fig. 20 Shows the formulas of some chemical compounds in methanol eluate. There are small peaks that are not recognised by the device.

## 4 Conclusions

First use of isopropanol as a solvent for extraction. This method of separation and isolation was used for the first time in this study and had not been used previously. This method allowed the isolation of a greater number of chemical compounds with less solvent consumption. The leaves of *Ailanthus glandulosa* contain effective constituents in good quantity, and the percentage of isopropanol extract was 9% of the weight of the sample. The main constituents of the extract were determined and these compounds were found to be esters, aldehydes, ketones, alcohols, carboxylic acids and a number of silicone compounds.

There will be a study of the effect of the isolated components on a number of bacteria and fungi. Taking care of this tree to benefit from its important components in many industrial fields.

## Author contribution statement

Sawsan Saad: Conceived and designed the experiments; Performed the experiments; Analyzed and interpreted the data; Contributed reagents, materials, analysis tools or data; Wrote the paper.

## Data availability statement

Data included in article/supp. Material/referenced in article.

## Additional information

There is supplemental content related to this article.

## Declaration of competing interest

The authors declare that they have no known competing financial interests or personal relationships that could have appeared to influence the work reported in this paper.
