# Supplementary material for: Isolation of the isopropanol extract components of the leaves of Ailanthus glandulosa by a new method
Source: Heliyon. 2023 Apr 30;9(5):e15881. doi: 10.1016/j.heliyon.2023.e15881 (PMC10192775; doi:10.1016/j.heliyon.2023.e15881)
Supplement: Multimedia component 1 [file mmc1.docx]

**Isolation of the isopropanol extract components of the leaves of *Ailanthus* *glandulosa* by a new method**

Sawsan Youseff Saad

Faculty of Dentistry, Manara University, Lattakia, Syria

**supplemental content**

**Mass spectra of some chemical compounds and fragmentation mechanics of the compounds**

1. **GC-MS chromatograms of eluate samples (B, C, D)**


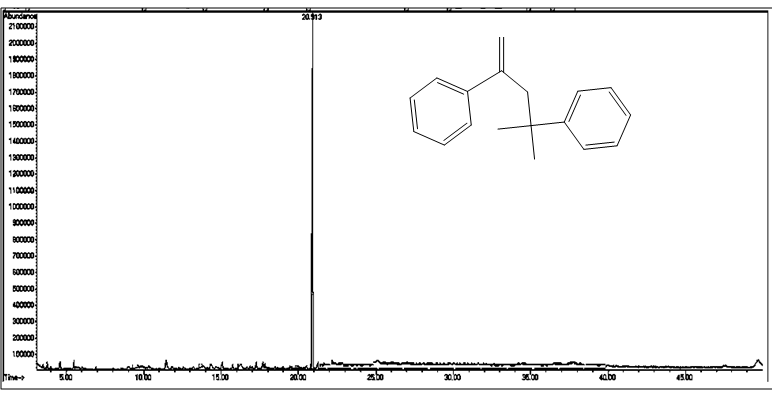


GC/MS chromatogram of sample B_1_ from dichloromethane eluate


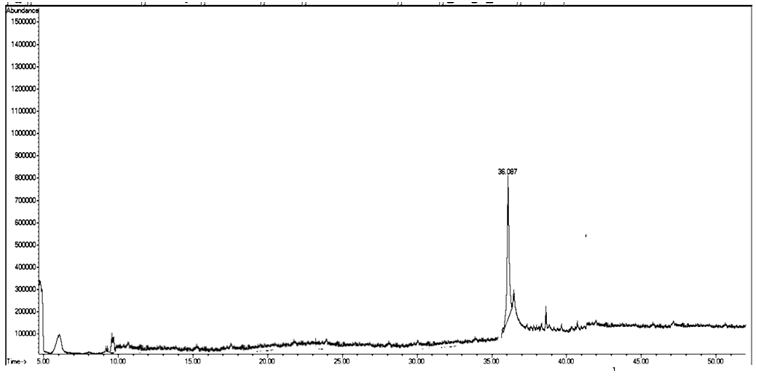


GC/MS chromatogram of sample B_2_ from dichloromethane eluate


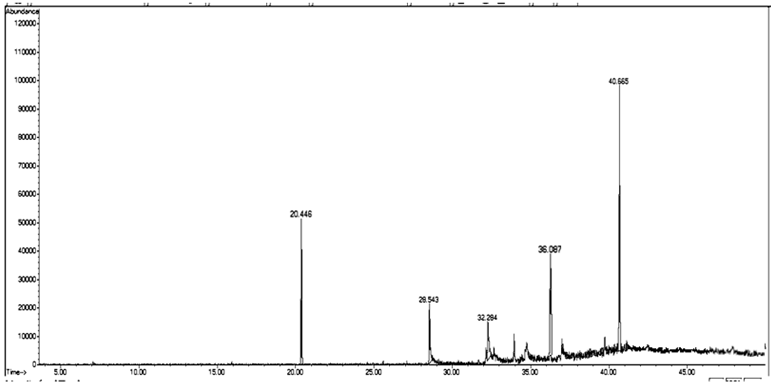


GC/MS chromatogram of sample B_3_ from dichloromethane eluate


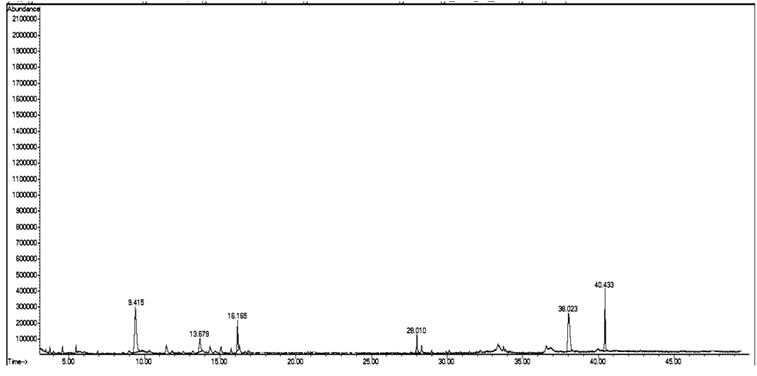


The GC/MS chromatogram of sample (C_1_) from the chloroform eluate


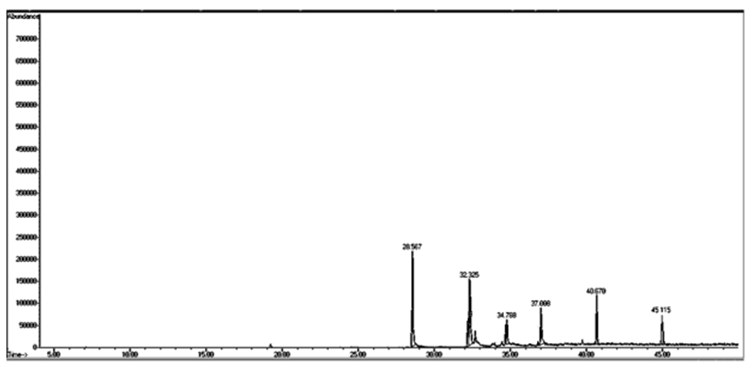


The GC/MS chromatogram of sample (C_2_) from the chloroform eluate


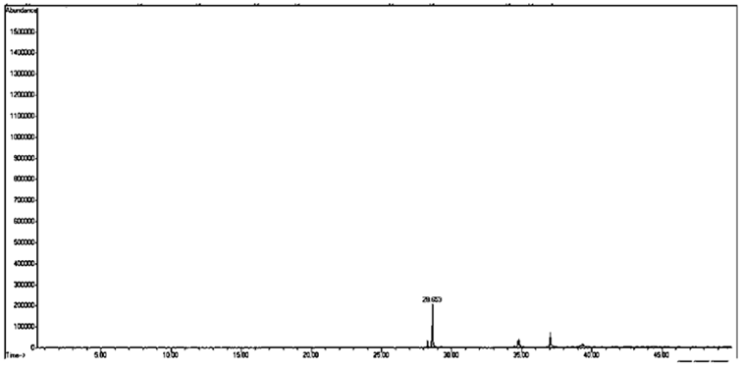


The GC/MS chromatogram of sample (C_m_) from the chloroform eluate


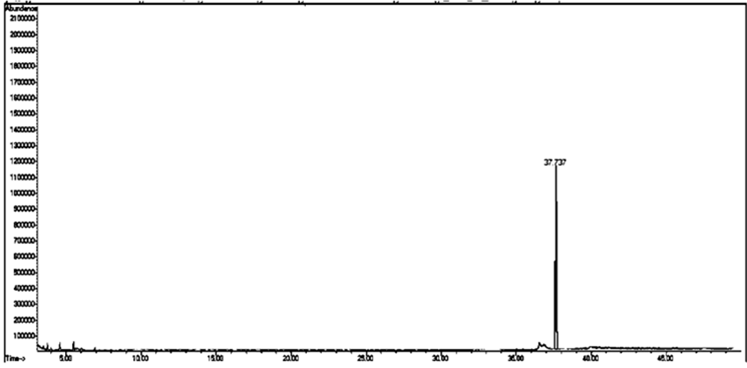


The GC/MS chromatogram of sample (C_n_) from the chloroform eluate


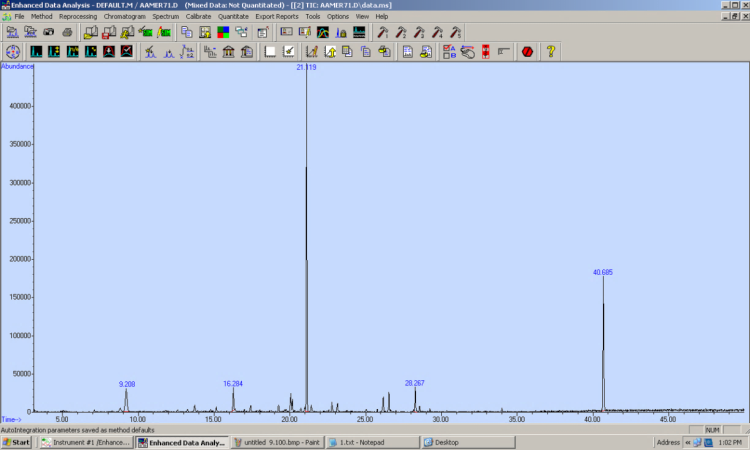


The GC/MS chromatogram of sample (C_3_) from the chloroform eluate


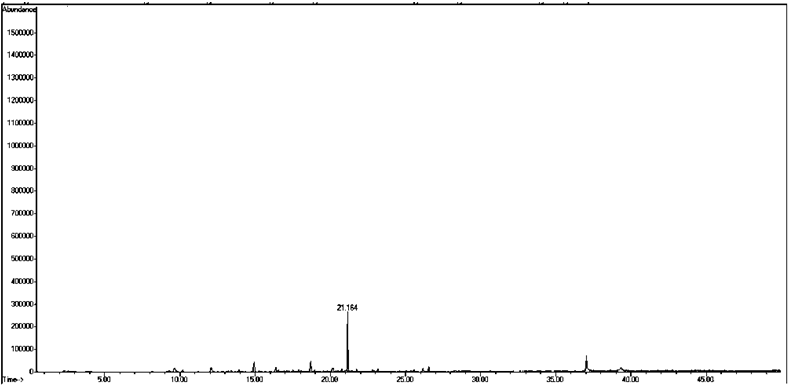


The GC/MS chromatogram of sample (C_3t_) from the chloroform eluate


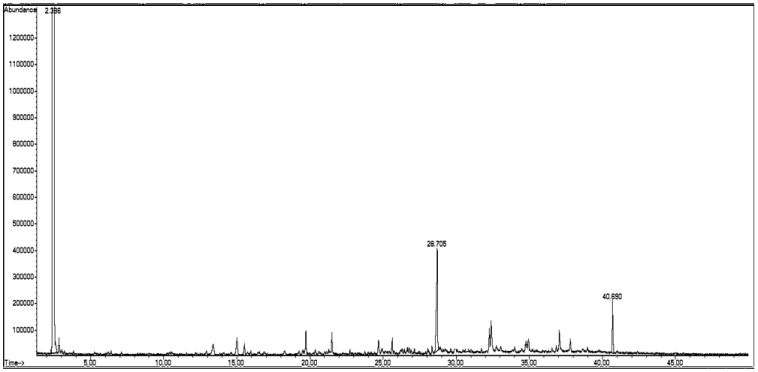


The GC/MS chromatogram of sample (D_1_) from the methanol eluate


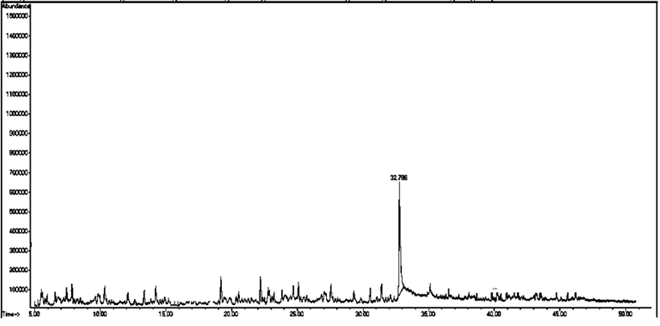


The GC/MS chromatogram of sample (D_2_) from the methanol eluate


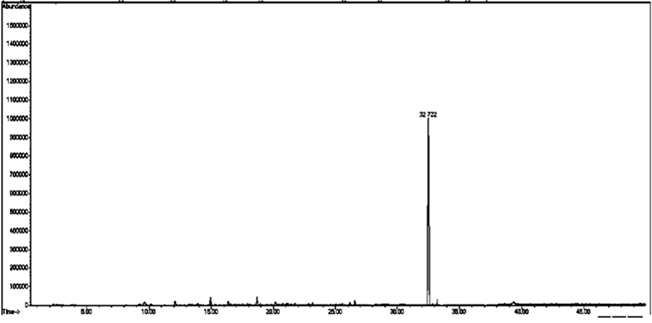


The GC/MS chromatogram of sample (D_3_) from the methanol eluate


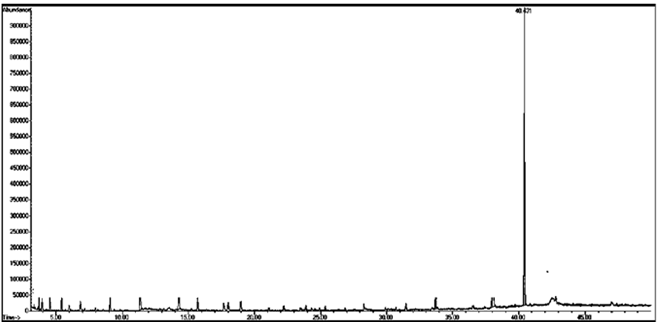


The GC/MS chromatogram of sample (D_4_) from the methanol eluate


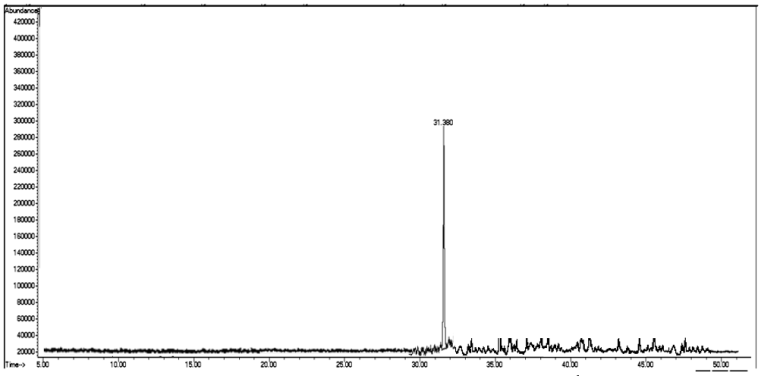


The GC/MS chromatogram of sample (D_5_) from the methanol eluate


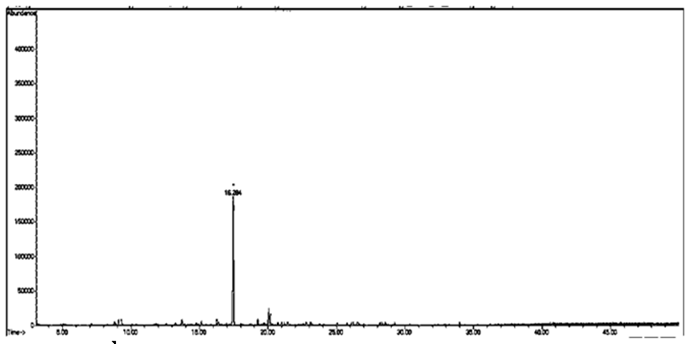


The GC/MS chromatogram of sample (D_6_) from the methanol eluate


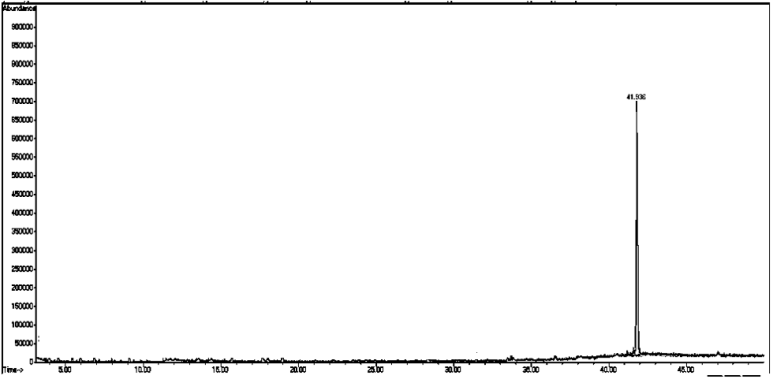


The GC/MS chromatogram of sample (D_7_) from the methanol eluate

**2- Mass spectra and fragmentation mechanisms of the compounds**


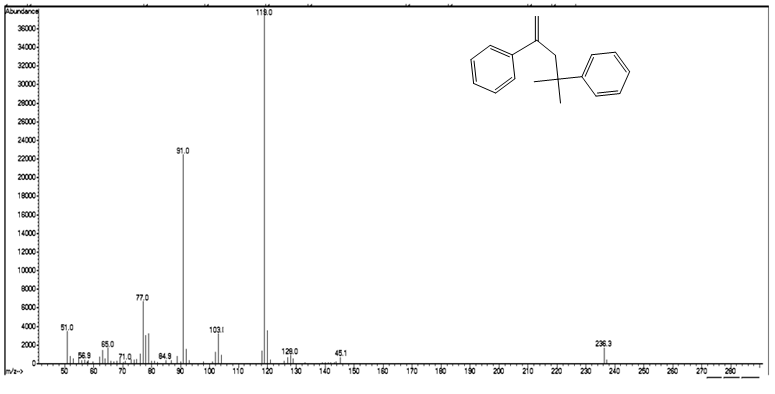


Fig 1. Mass spectrum of 2,4-Diphenyl-4-Methyl-1-Pentene (B_1_)


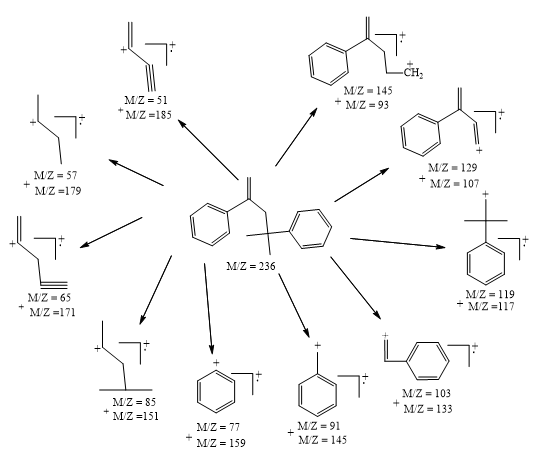


Fig 2. The mechanism of fragmentation of compound 2,4-Diphenyl-4-Methyl-1-Pentene


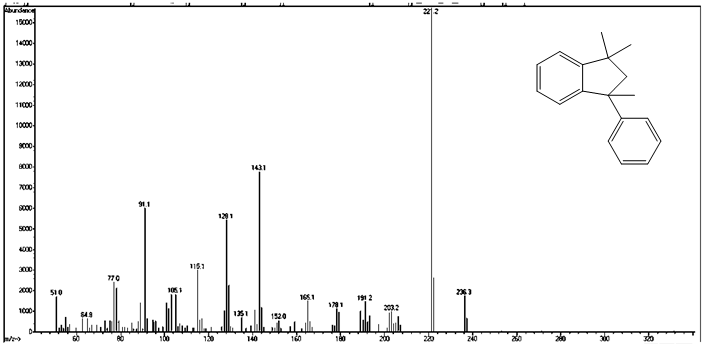


Fig 3. Mass spectrum of 1,3,3-trimethyl-1-phenyl Indane (B_2_)


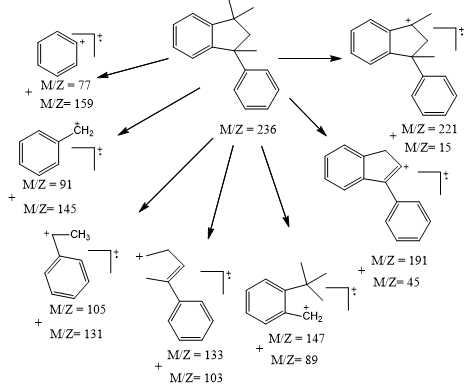


Fig 4. The mechanism of fragmentation of compound 1,3,3-trimethyl-1-phenyl Indane


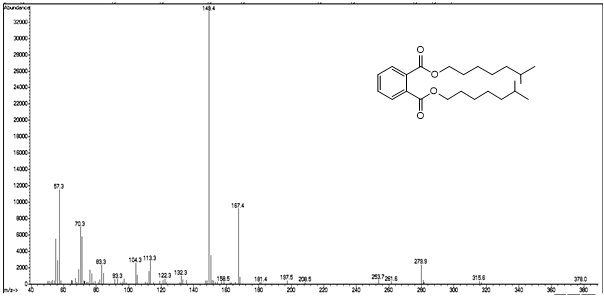


Fig 5. Mass spectrum of 1,2-Benzene dicarboxylic acid diisooctylester (B_3d_)


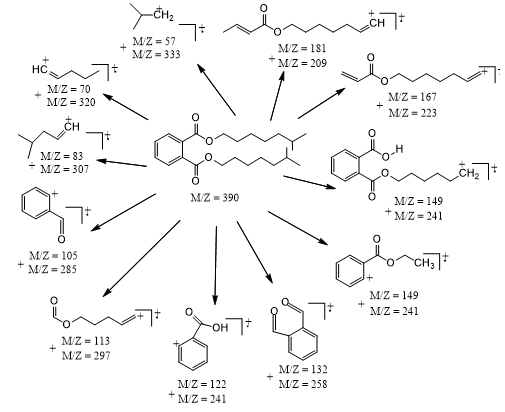


Fig 6. The mechanism of fragmentation of compound 1,2-Benzene dicarboxylic acid diisooctylester (B_3d_)


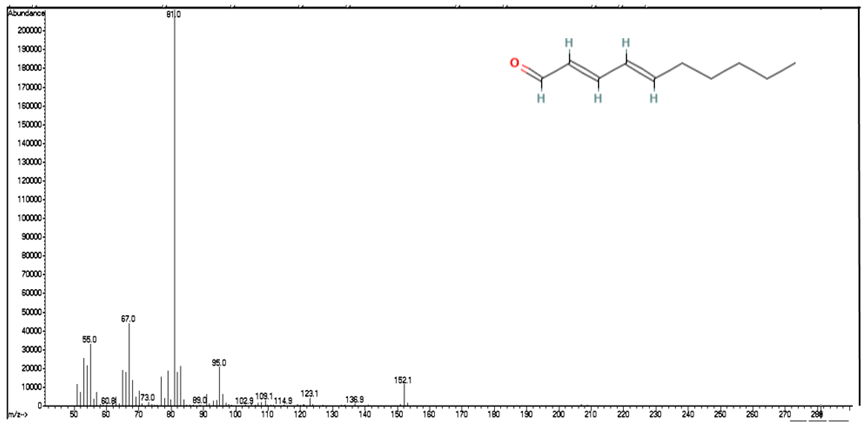


Fig 7. Mass spectrum of (E,E) 2,4-Decadienal (C_1a_)


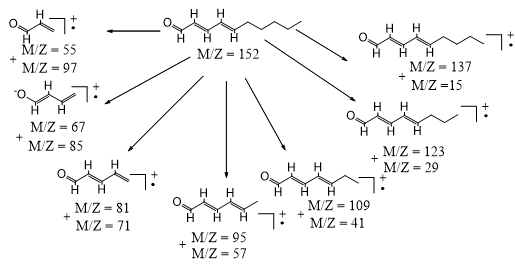


Fig 8. The mechanism of fragmentation of compound (E,E) 2,4-Decadienal (C_1a_)


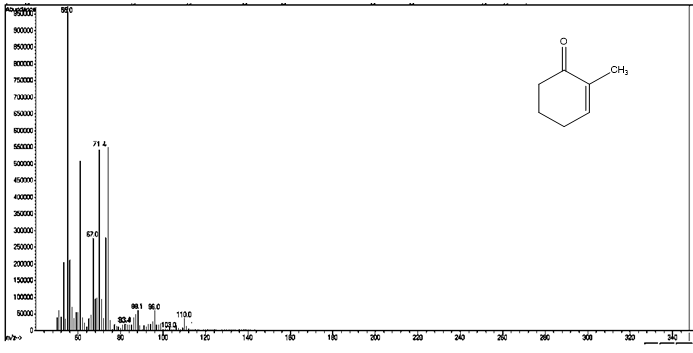


Fig 9. Mass spectrum of 2-Methyl-2-Cyclohexenone (C_1c_)


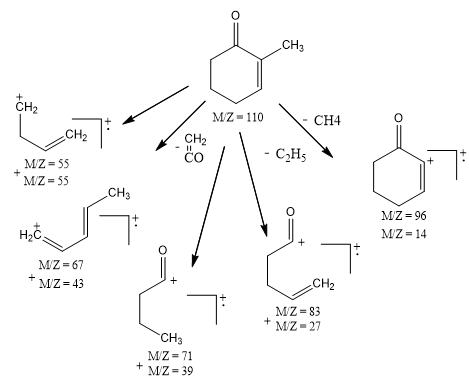


Fig 10. The mechanism of fragmentation of compound 2-Methyl – 2 - Cyclohexenone (C_1c_)


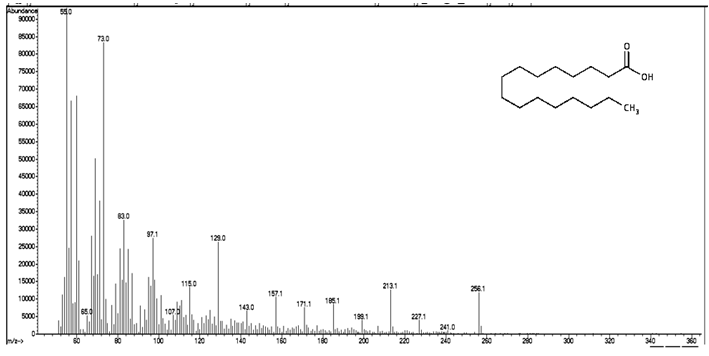


Fig 11. Mass spectrum of n-Hexadecanoic acid (C_2b_)


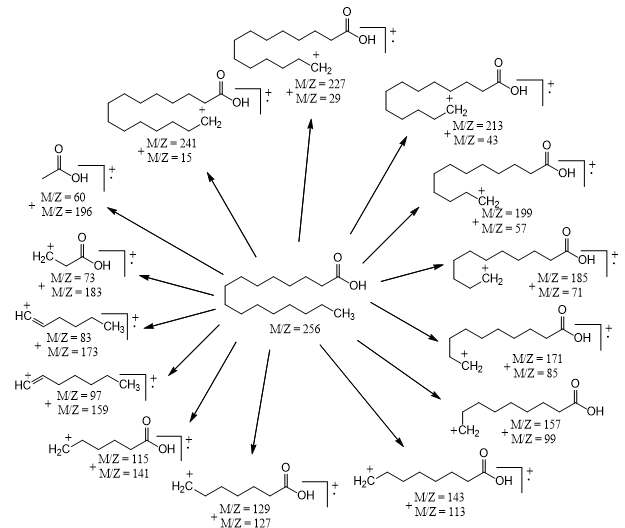


Fig 12. The mechanism of fragmentation of compound n-Hexadecanoic acid (C_2b_)


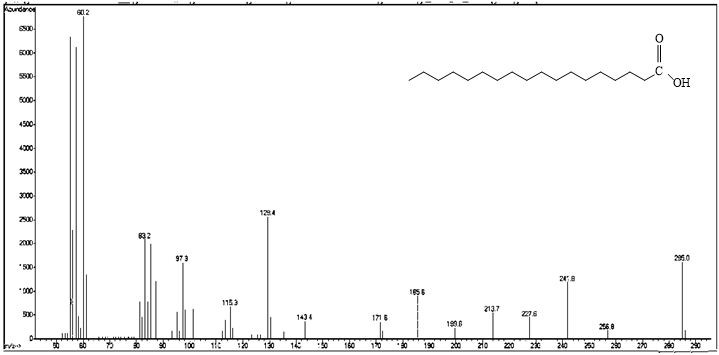


Fig 13. Mass spectrum of Octadecanoic acid (C_2d_)


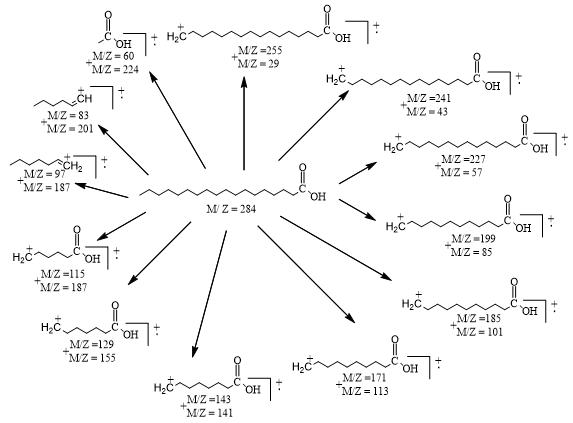


Fig 14. The mechanism of fragmentation of compound Octadecanoic acid (C_2d_)


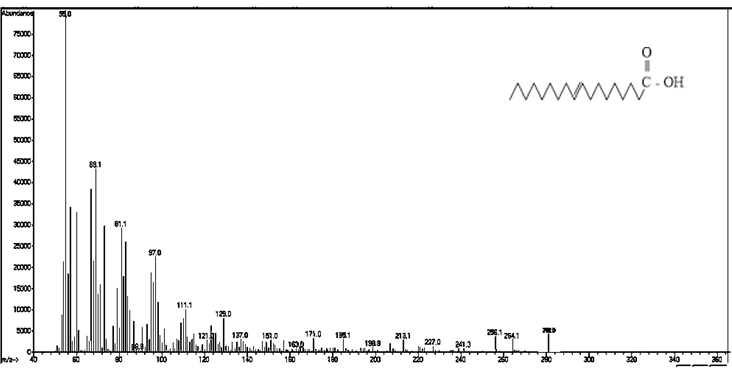


Fig 15. Mass spectrum of (Z) 9-Octadecenoic acid (C_2h_)


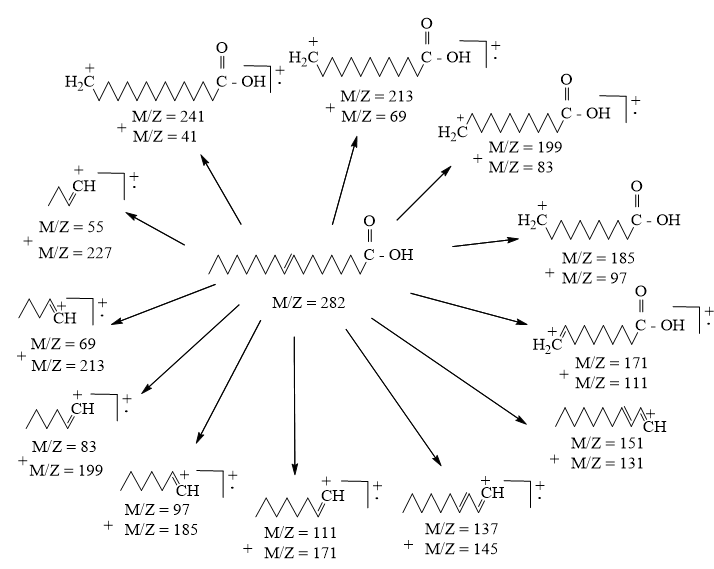


Fig 16. The mechanism of fragmentation of compound (Z) 9-Octadecenoic acid (C_2h_)


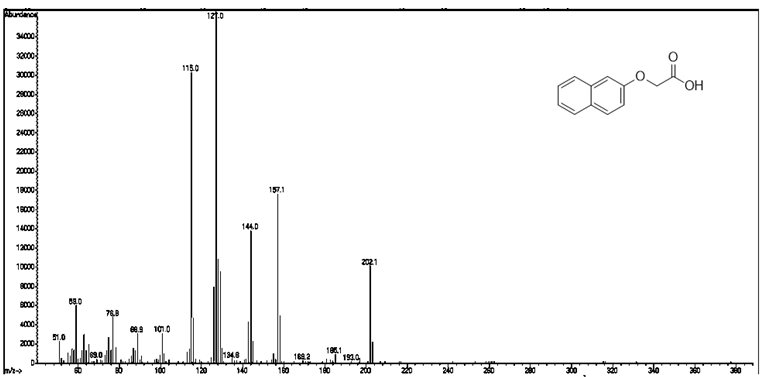


Fig 17. Mass spectrum of 2-Naphthoxy acetic acid (C_m_)

Fig 18. The mechanism of fragmentation of compound 2-naphthoxy acetic acid (C_m_)


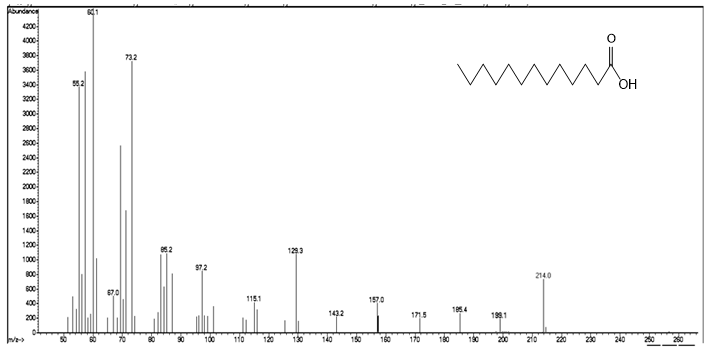


Fig 19. Mass spectrum of Tridecanoic acid (C_n_)


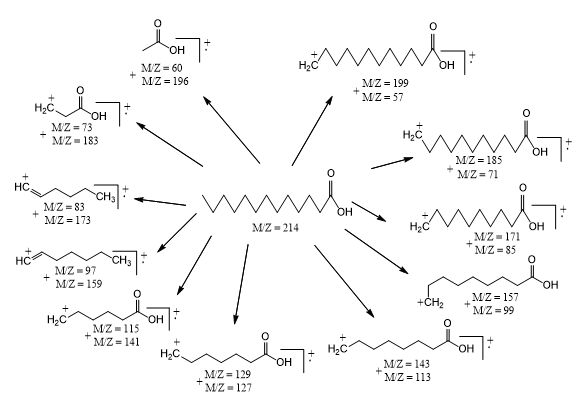


Fig 20. The mechanism of fragmentation of compound Tridecanoic acid (C_n_)


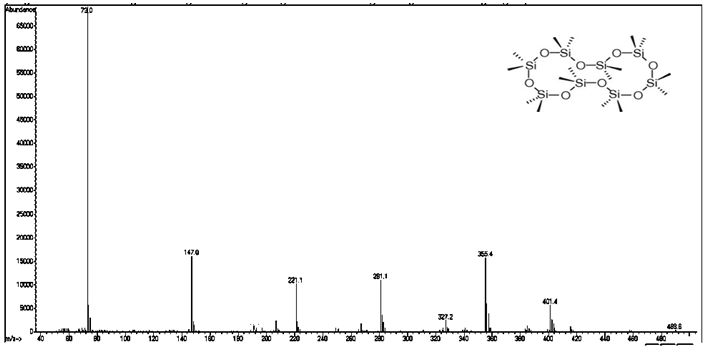


Fig 21. Mass spectrum of Hexadecamethyl cyclooctasiloxan (C_3t_)

Fig 22. The mechanism of fragmentation of compound Hexadecamethyl cyclooctasiloxan (C_3t_)


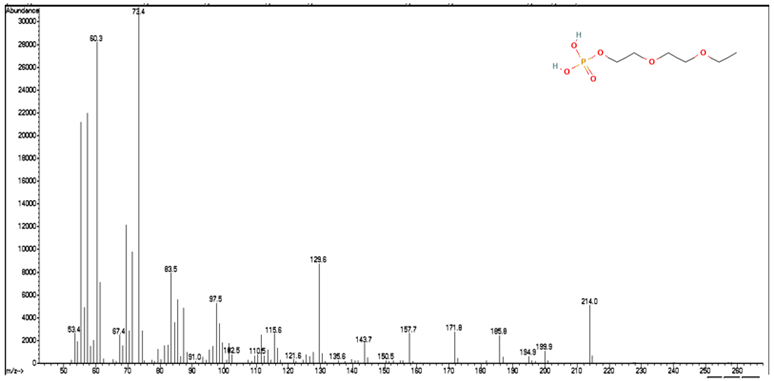


Fig 23. Mass spectrum of 2-(2-ethoxyethoxy) phosphate ethanol (D_1a_)


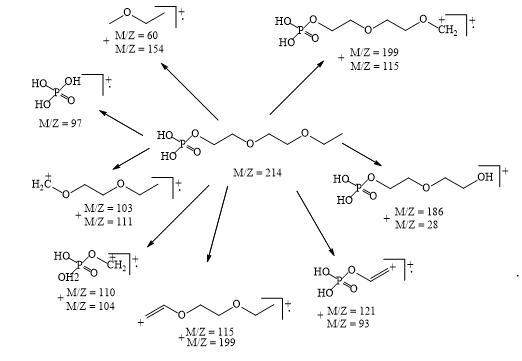


Fig 24. The mechanism of fragmentation of compound 2-(2-ethoxyethoxy) phosphate ethanol (D_1a_)


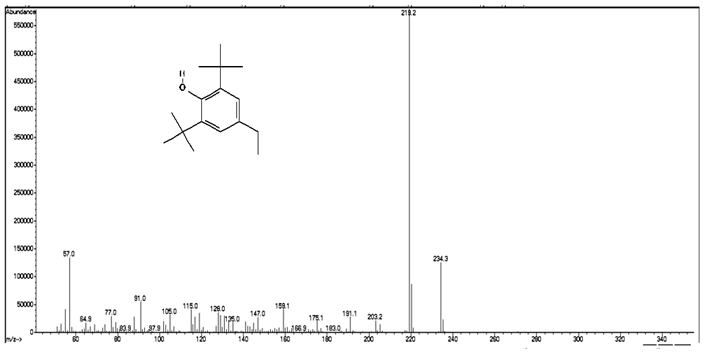


Fig 25. Mass spectrum of 2,6*-*bis (1*,*1*-*Dimethylethyl)-4-ethyl Phenol (D_2_)


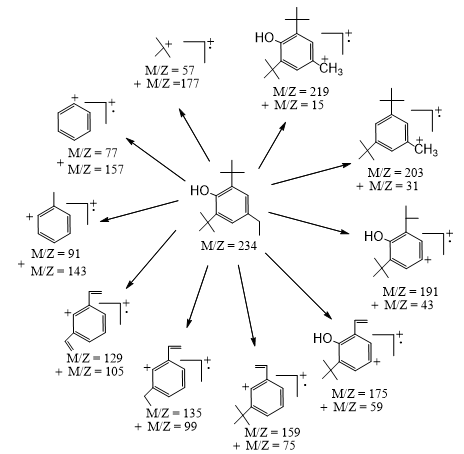


Fig 26. The mechanism of fragmentation of compound 2,6*-*bis (1,1*-*Dimethylethyl)-4-ethyl Phenol (D_2_)


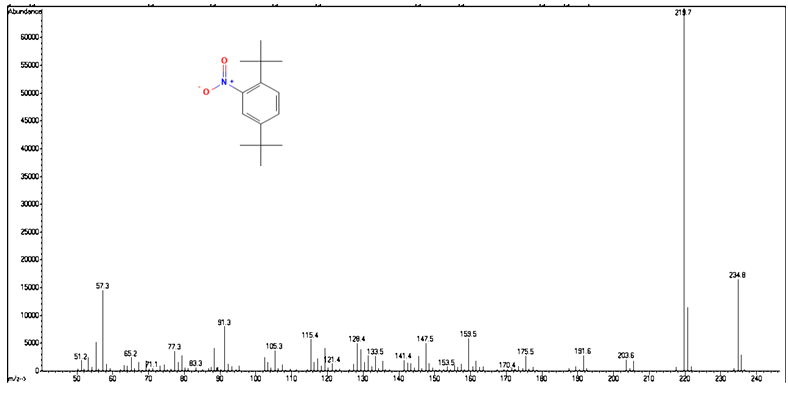


Fig 27. Mass spectrum of 2,5-di-tert-Butylnitrobenzene (D_3_)


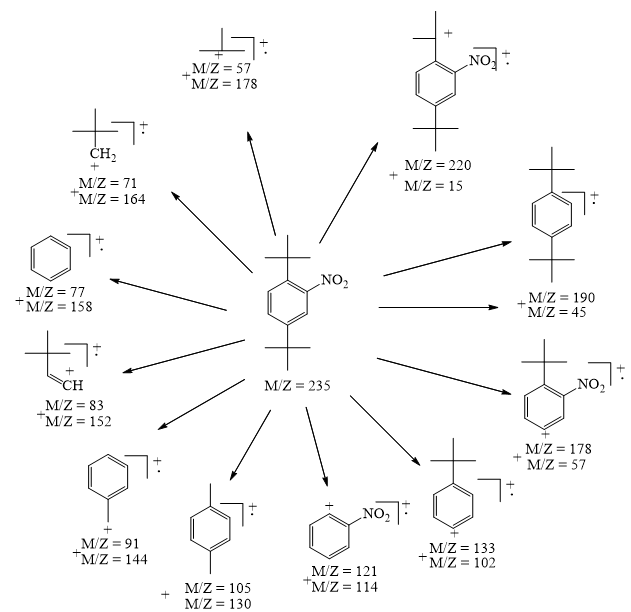


Fig 28. The mechanism of fragmentation of compound 2,5-di-tert-Butylnitrobenzene (D_3_)


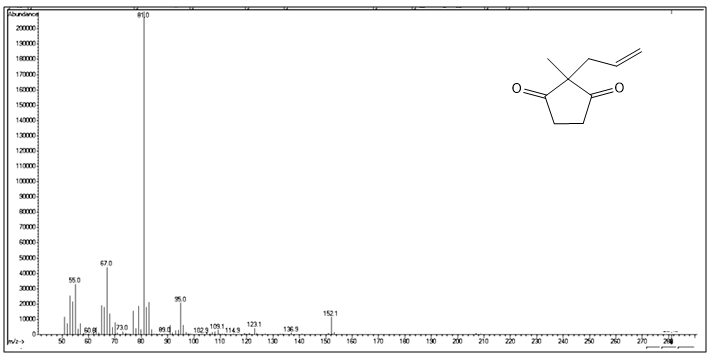


Fig 29. Mass spectrum of 2-Allyl-2-methyl-1,3-cyclopentanedione (D_4_)


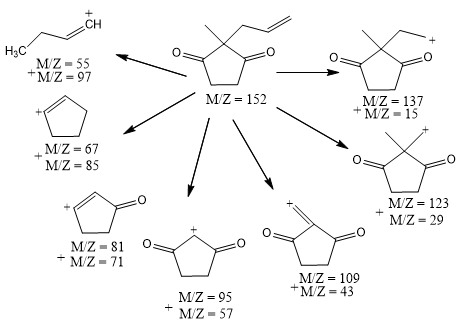


Fig 30. The mechanism of fragmentation of compound 2-Allyl-2-methyl-1,3-cyclopentanedione (D_4_)

.


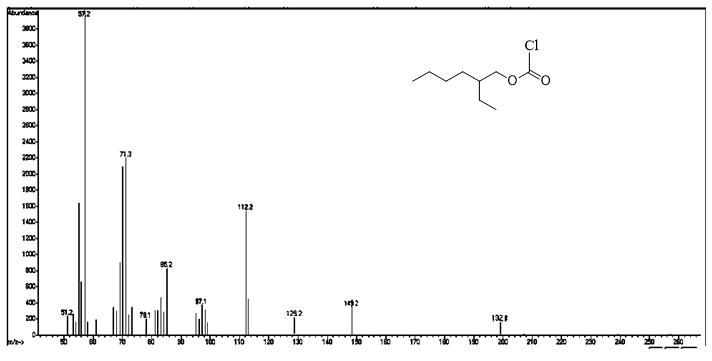


Fig 31. Mass spectrum of 2-Ethylhexylchloroformate (D_5_)


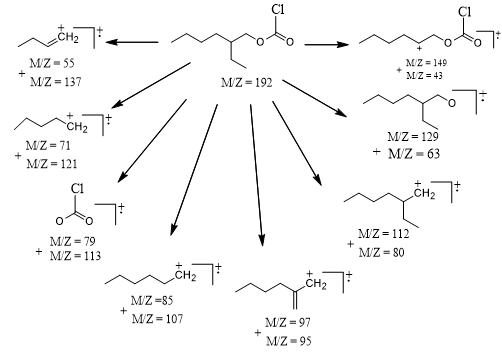


Fig 32. The mechanism of fragmentation of compound 2-Ethylhexylchloroformate (D_5_)


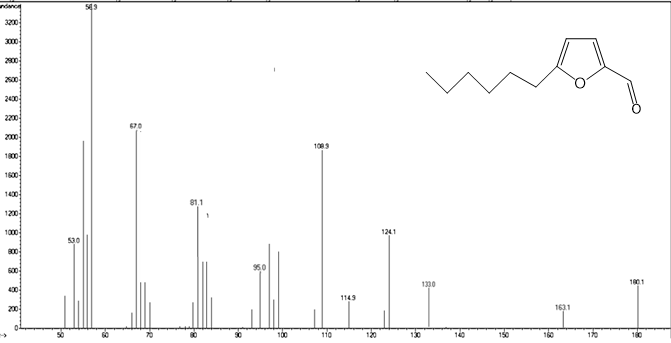


Fig 33. Mass spectrum of 5-Hexyl-2-furaldehyde (D_6_)

Fig 34. The mechanism of fragmentation of compound 5-Hexyl-2-furaldehyde (D_6_)


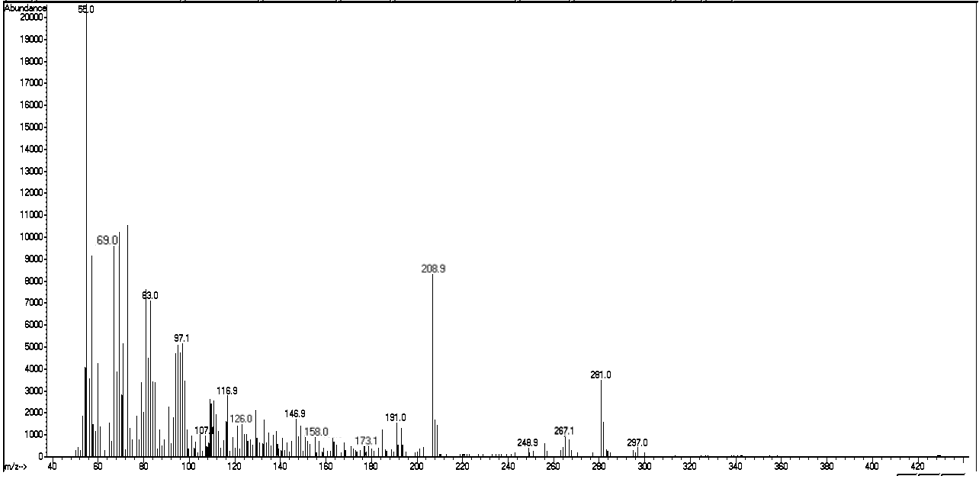


Fig 35. Mass spectrum of 16-Nitrobicyclo [10.4.0] hexadecan-1-ol-13-one (D_7_)

Fig 36. The mechanism of fragmentation of compound 16-Nitrobicyclo [10.4.0] hexadecan-1-ol-13-one (D_7_)
